# Factors associated with patient recall of key information in ambulatory specialty care visits: Results of an innovative methodology

**DOI:** 10.1371/journal.pone.0191940

**Published:** 2018-02-01

**Authors:** M. Barton Laws, Yoojin Lee, Tatiana Taubin, William H. Rogers, Ira B. Wilson

**Affiliations:** 1 Brown University School of Public Health, Department of Health Services, Policy and Practice, Providence, Rhode Island, United States of America; 2 Tufts Medical Center, Institute for Clinical Research and Health Policy Studies, Boston, Massachusetts, United States of America; Public Library of Science, UNITED KINGDOM

## Abstract

While some studies have assessed patient recall of important information from ambulatory care visits, none has done so recently. Furthermore, little is known about features of clinical interactions which are associated with patient understanding and recall, without which shared decision making, a widely shared ideal for patient care, cannot occur. Our objective was to evaluate characteristics of patients and outpatient encounters associated with patient recall of information after one week, along with observation of elements of shared decision making. This was an observational study based on coded transcripts of 189 outpatient encounters, and post-visit interviews with patients 1 week later. Coding used three previously validated systems, adopted for this study. Forty-nine percent of decisions and recommendations were recalled accurately without prompting; 36% recalled with a prompt; 15% recalled erroneously or not at all. Provider behaviors hypothesized to be associated with patient recall, such as open-questioning and “teach back,” were rare. Patients with less than high school education recalled 38% of items freely and accurately, while patients with a college degree recalled 65% (p < .0001). In a multivariate model, the total number of items to be recalled per visit, and percentage of utterances in decision-making processes by the provider (“verbal dominance”), were significant predictors of poorer recall. The item count was associated with poorer recall for lower, but not higher, educated patients.

## Introduction

The “patient centered” care movement in the 1970s[1] led to interest in models of shared decision making[2] or concordance.[3] Shared decision making defines the goal of clinical communication as agreement between physicians and patients about whether, when, and how medicines or other interventions are to be taken, via discussion that includes and respects the patient’s beliefs and wishes.[4, 5] Barry argues that when there are substantial tradeoffs among potential benefits, risks, and burdens of treatment, informed choice by patients is essential to optimizing outcomes.[6] Patient satisfaction has also been associated with directly observed provider communication behaviors,[7–10] and with patient reports of engagement and autonomy in medical care.[7, 11–13] Patients reporting more engagement and autonomy have in turn been found to be more adherent to therapy.[14, 15]

However, available evidence shows that discussions around decisions in routine outpatient care lack essential components of informed choice;[16] as do decisions about major elective vascular surgery.^6^ Physicians often do not elicit patients’ preferences or discuss reasons not to take action;[17, 18] disagreement on diabetes management goals and strategies is common;[19] and physician-patient communication about medications is poor when new medications are started,[20] when patients are non-adherent,[21] and when patients make medication changes on their own.^14,15^

While many factors affect whether decision making is shared and informed, and affect adherence to medication regimens and other medical advice, people who do not properly understand and remember instructions have no chance to be adherent. Nor can patients who do not correctly understand the information provided by their physician legitimately be said to have shared in decision making.

Studies over several decades have consistently found that patients do not correctly recall much of the recommendations and information given by their physicians.[22–26] Typically about half of items are found to be accurately recalled.

Several communicative behaviors are believed or hypothesized to be associated with better recall, in medical and other contexts. One is summarization at the conclusion of the encounter.[27, 28] Another widely recognized method is “teach back,” asking the patient to repeat the information in his or her own words.[29, 30] Providing structure for information in the form of an initial “table of contents” followed by “chapter headings” has been found to improve recall of medical information in an experiment with college students.[31] However, these have not generally be evaluated in the context of ambulatory health care, either for prevalence or effectiveness.

Knowledge alone is not sufficient for patients to effectively participate in decision making.[32] While various models of shared decision making have been proposed, they have been found to share the requirement that providers inform the patient about the nature of the decision and available options; elicit patient preferences; and integrate those preferences into a decision making process.[5, 33] Accordingly, we set out to assess the extent to which providers use behaviors hypothesized to promote recall in ordinary clinical interactions, to assess the extent to which elements of shared decision making are present, and to determine predictors of accurate recall and understanding by patients.

## Methods

### Subjects

We conducted an observational study at two hospital-based outpatient cardiology clinics and one hospital-based outpatient nephrology clinic. Both providers and patients gave written informed consent to participate, as did any people accompanying patients who were present for the encounter. Data collection occurred during 2013 and 2014. Clinic staff informed us that first visits tend to be uneventful, consisting largely of evaluation and orientation. Therefore, we enrolled patients who were either coming for second visits, or who were experiencing an exacerbation or significant treatment decision; and who were scheduled to see an enrolled provider. Clinic personnel informed study staff ahead of time when an eligible patient was expected, so a Research Assistant (RA) could be present. Reception staff or a research nurse asked patients if they were interested in the study, and referred interested patients and any companions to the RA for enrollment. This study was approved by the Institutional Review Boards of both the health care system where the data was collected, and the university where the principal work was performed.

### Data collection

Participants completed a brief demographic background questionnaire. The RA then placed an inconspicuous digital recorder in the examining room, and turned it on. Providers were instructed how to turn it off if they or the patient wished. Upon completion of the visit, the RA retrieved the recorder. In the next few days, RAs prepared a structured abstract of the visit using spreadsheet software, listing treatment options, recommendations and decisions discussed in the visit; important information discussed in the visit; and behavioral or lifestyle recommendations. Based on prior consultation with providers, the abstract form was pre-populated with common treatments in the respective specialties, such as salt restriction or commonly used medications, with the ability to add any other information freely.

An RA then made a pre-arranged phone call to the patient approximately 7 days after the visit. The RA first asked the patient to freely recall any important information, treatment options, decisions or rationale discussed in the visit. For anything the patient failed to mention, the RA would probe using the structured abstract. There followed a semi-structured discussion of the patient’s experience of the visit, including any unanswered questions or concerns that were not addressed. (The RA also administered structured questionnaires which are not discussed in this report.) If patients had return visits during the study period, we also collected data for those visits, yielding longitudinal information for some participants. An outside service transcribed each visit. We corrected the transcripts and then coded them using three systems.

### Transcript coding

The first coding system used is the Generalized Medical Interaction System (GMIAS), which was designed to extend extant systems for coding and analyzing provider-patient communication. These systems, which are generally based on defining various physician and patient verbal behaviors and counting their frequencies, have produced insight into physician and patient role relationships, and have described relationships between physician and patient characteristics and a variety of relevant outcomes.[15, 34, 35] However, most coding systems lack a guiding theoretical framework,[36] and assign only a single code to each utterance. The GMIAS is based on speech act theory, a central construct in sociolinguistics, which refers to the social act embodied in an utterance, such as questioning, requesting, directing, representing facts, or expressing wishes.[37, 38] It has been described previously.[39] The GMIAS assigns two codes–a speech act code and a topic code—which enables separate or conjoint analysis of verbal behavior and content.[40] The unit of analysis is a completed speech act. While we do not use topic codes in this analysis, we do consider specific speech acts including various forms of question–open, closed and leading; and forms of knowledge check–non-specific (e.g. “Do you understand?”) and teach-back. “What do you want to do” is an open question. Consistent with good counseling practice, “Do you have any questions” is considered a closed question, while “What questions do you have?” is an open question. The GMIAS is designed to be readily adaptable to any clinical practice question or situation, while retaining its basic structure to permit comparability.

The second coding system we used is the Comprehensive Analysis of the Structure of Encounters System (CASES).[41] CASES extends the capabilities of the GMIAS by parsing the structure of the encounter at a higher level. It firsts identifies each of the problems, issues or tasks addressed in the encounter, which we call “threads,” each of which may contain any or all of 4 “processes,” which we label Presentation, Information, Engagement, and Resolution. Presentation is development of information specific to the patient’s situation, such as symptom reports, history taking, test results and clinical examination; Information pertains to more general facts such as the etiology of a disease or typical prognosis; Engagement refers to interpersonal interactions such as expression of empathy and support; and Resolution is the decision making leading to an outcome. Information that is adduced during the Presentation or Information processes may subsequently enter a Resolution process if it is specifically referenced during discussion or announcement of a decision. Threads are also coded as to the nature of the resolution, e.g. take no action, prescribe a drug, make a referral, or commit to a lifestyle change. We also classified resolutions as pertaining to medical intervention, such as prescribing a medication, changing a dosage, or deciding on a procedure; or behavioral or lifestyle changes or maintenance such as diet, physical activity, or adherence to a previously prescribed regimen. Not all threads result in any action. There may be no perceived need because, for example a condition is self-resolving, a finding is negative, or the thread is merely updating the provider on a situation. Sometimes, however, a problem is simply not addressed.

The CASES coding enabled us to match patient’s recall of individual decisions and recommendations to the resolution processes in which they were discussed. Note that there can be more than one resolution to a thread, e.g. change in the dose of one medication, introduction of a new one, and a dietary modification (producing two medical resolutions and one behavioral resolution). In addition to threads pertaining to specific problems or issues, CASES codes for initial agenda setting, and “wrap up” at the end of the visit, reviewing key decisions.

There were three coders for this study. One was experienced in GMIAS and CASES coding prior to this study. She was the “gold standard” coder. After initial didactic training, other coders coded some of the same encounters as the gold standad and the team met several times to discuss and resolve discrepancies. We then computed intercoder reliability for both double coding using both systems. For the GMIAS, with topic codes adapted to the specialties, in repeated intercoder reliability tests we achieved kappa statistics for top-level topic codes consistently above .86 and as high as .98, with similar results for speech act codes. Kappa statistics of 0.61–0.80 are considered ‘‘substantial.”[42] For the CASES, there was 85% agreement on assignment of utterances to threads, 85–100% agreement on assignment of processes within threads, and 100% agreement on thread ownership in early tests. As there is necessarily some judgment involved in assigning utterances to threads, however, coders would discuss difficult issues and resolve them by consensus.

The third coding system we used is the OPTIONS system, a checklist for elements of shared decision making.[43] Examples of OPTIONS codes are “states the existence of more than one option to deal with the problem,” and “Pros and cons of options.” We modified the OPTIONS system to collapse levels of strength for some items, in order to improve reliability. The OPTIONS system was originally developed to focus on a single index problem.[43] Routine outpatient visits, however, may produce any number of individual decisions. The CASES system created a structure for OPTIONS coding of each treatment decision, making it straightforward to apply the system to encounters with multiple decision outcomes.

### Dependent variable

The dependent variable in our analyses was recall quality. We matched the transcripts of the follow-up interviews with the corresponding visits, and coded patients’ recall for each resolution item according to two dimensions: whether it was recalled freely, recalled only after prompting, or not at all; and whether recall quality was entirely erroneous, partially correct, or essentially correct. We used this coding to classify items as recalled freely and accurately; recalled accurately with prompting; or recalled inaccurately or not at all.

Note that information is often a component of a resolution. For example, a diagnosis may constitute a resolution, or the information that a test result is not sufficiently concerning to warrant further action, or the converse. Resolutions may consist of instruction on how to do something, or may consist of information if the presenting problem was that the patient wanted information or explanation. Additionally, our assessment of the accuracy of recall of treatment decisions included the patient’s understanding of the rationale. So generally, recall of the information relevant to decision making, or at least explicitly referencd in decision making, was assessed. Most of the remaining information flow in encounters is actually from patient to clinician, as the clinician takes history and investigates problems. There is some additional information flow captured in what we call the information process, such as prognostic and etiological information. If this figures in decision making, it will usually be incorporated in assessment of accurate understanding.

### Analyses

We conducted analyses using SAS, version 9.4 (SAS Institute, Cary, North Carolina). We first generated descriptive data on the frequency of items codable for recall, in both medical and behavioral resolutions; the quality of patient recall; the prevalence of communicative behaviors hypothesized to be associated with better recall; and OPTIONS codes. As most of the provider behaviors were uncommon, as were OPTIONS codes, we could not test the association with recall.

We tested the bivariate association of recall quality with patient level of formal education using chi-square; and the number of items to be recalled within a visit, and the ratio of provider to patient utterances (speech acts) in the resolution processes within a visit using ordered logit models accounting for clustering within patients.

We constructed multivariate ordered logit models, accounting for clustering within patients to predict patient recall using patient education, number of items to be recalled in a visit, and the ratio of provider to total (patient + provider) utterances in resolutions processes within the visit. We tested patient age, gender, and race ethnicity (white non-Hispanic vs. other) as covariates, and we constructed separate models for behavioral and medical resolutions. Covariates that were not significant at a p<0.05 level were dropped from the model.

**Fig 1:**
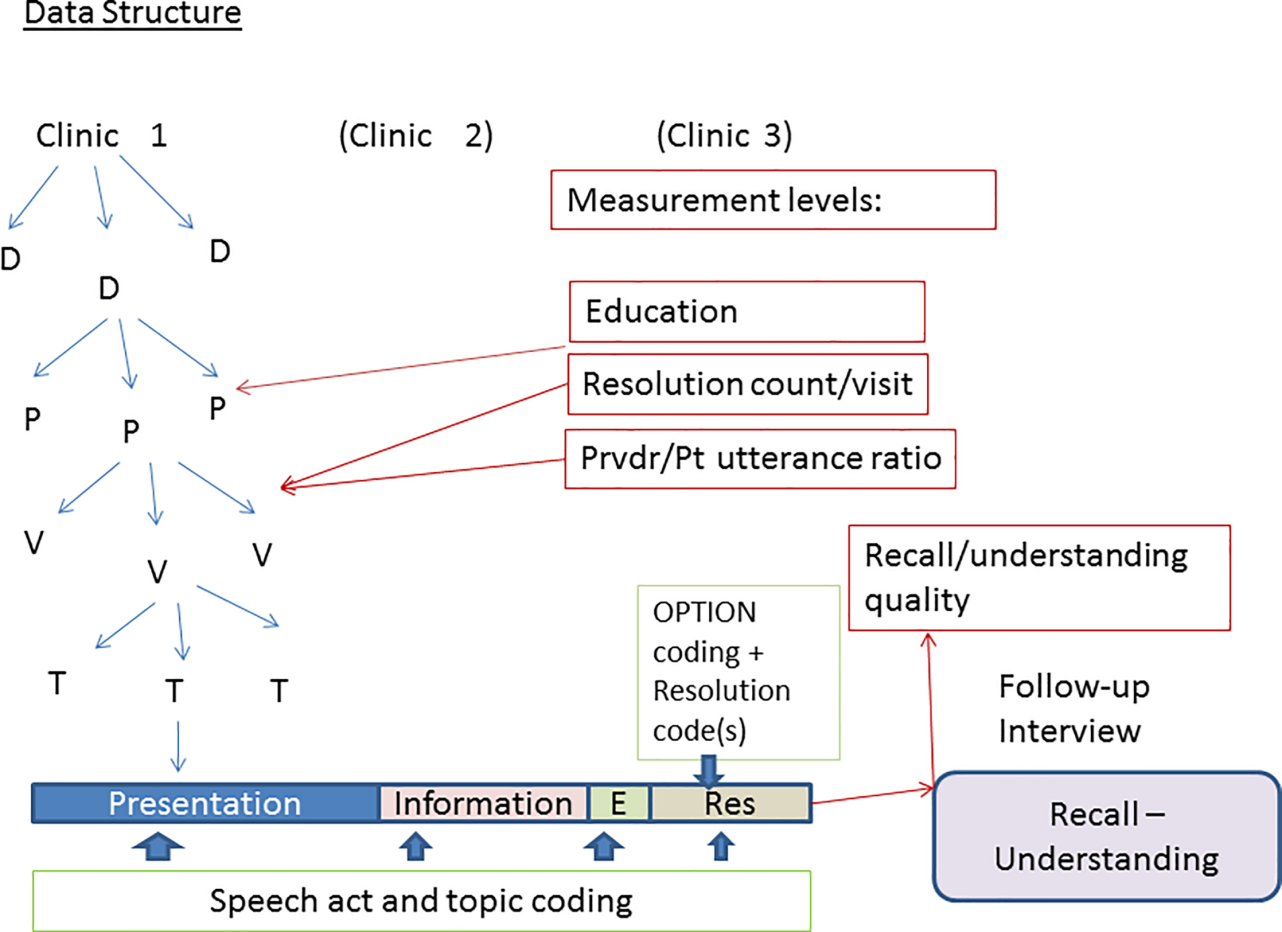
Data structure.

Fig 1 shows the structure of the data, with resolutions nested within visits, nested within patient, nested within providers, nested within clinics. The dependent variable was measured at the level of the individual resolution/recall; ratio of provider to patient utterances and number of items to be recalled measured at the level of the visit; and patient education at the patient level.

### Stakeholder engagement

We consulted with physicians in the participating clinics to inform the research design, including the topic coding schemes and structured abstracts for nephrology and cardiology. We also organized a standing consultative group of five nephrology and cardiology patients, plus one patient spouse, with which we consulted on study design, analysis and interpretation of results. Finally, we presented these results for discussion in a symposium to a diverse group of physicians, patients, and ancillary providers such as case managers and behavioral counselors (Providence, RI, January 9, 2016). Results of the symposium inform our interpretation (see Discussion).

## Results

### Participants

In one cardiology clinic we enrolled 4 providers; in the second cardiology clinic we enrolled 7 providers, and in the nephrology clinic we enrolled 8 providers. We approached a total of 130 patients, of whom 108 gave informed consent. At least one visit was recorded, and follow-up data obtained for 102. One of these was observed to have substantial cognitive impairment and so was excluded from the analysis, leaving a total of 101 patients. These patients completed a total of 189 visits with complete follow-up data. Patients ranged in age from 19 to 89, with a mean age of 57. There were 47 men and 54 women. Seventy-three of the 101 subjects were white, non-Hispanic. (See Table 1.) Visits occurred from September 2012 to March 2013.

**Table 1:** Patient and visit characteristics.

Characteristic	Value
**Patients (N = 101)**	
Age, mean (sd)	57.1 (15.9)
Female, N (%)	54 (53.5)
Race/Ethnicity, N (%)	
White	73 (72.3)
Black	20 (19.8)
Other	8 (7.9)
Center, N (%)	
HF	41 (40.6)
MC	27 (26.7)
RI	33 (32.7)
Education, N (%)	
7~11^th^ Grade	16 (15.8)
12^th^ Grade	29 (28.7)
1~3 year in College	29 (28.7)
College graduate or above	27 (26.7)
**Visits (N = 189)**	
Number of visits per patient	
1	101
2	48
3	27
4	13
Total number of threads*	1344
Threads per visit, mean (sd)	11.4 (2.7)
Threads with Medical process, N (%)	681 (51.0)
Threads with Behavioral process, N (%)	388 (29.1)
Resolution count/visit, mean (sd)	12.2 (6.4)
Fraction of utterances by provider, mean % (sd)	0.6 (0.1)

* “Threads” refers to each of the specific problems and issues discussed in the encounter; resolutions refers to decisions and recommendations made to address the issues in each thread. There may be multiple resolutions per thread.

### Recall opportunities, recall quality and recall promoting behaviors

As the follow-up interviews were based on review of audio recordings under time constraints, whereas the coding was based on careful review of transcripts, we found some items had been overlooked in the interviews. By definition, these were not recalled freely, meaning our overall assessment of recall quality is probably a slight over-estimate. Of the 12.6% of Medical resolutions considered codable for recall which had been overlooked in the follow-up interview, most of these were either a patient commitment to undergo a procedure elsewhere, a deferred decision, or a physician commitment to do something in the future. Slightly less than 15% of Behavioral resolutions were overlooked in the follow-up, most of them patient commitments to maintenance of a health-promoting behavior. These could have been considered out of the universe of relevant decisions, but we treated them as missing values, which we assume are randomly distributed.

Of 189 visits, all but one had at least one recall-coded medical decision or recommendation, with a mean of 4.9 per visit (range 1 to 16). At least one recall-coded behavioral resolution occurred in 149 of the 189 visits, with a mean of 2.7 per visit (range 1 to 14). The total of coded recalls was 917 medical resolutions and 402 behavioral resolutions. Overall, 49% of resolutions were recalled freely and accurately, and an additional 36% were recalled accurately with a prompt. Fifteen percent could not be recalled or were recalled erroneously. The numbers were similar when medical and behavioral resolutions were examined separately.

Most items that were recalled with or without prompting were recalled accurately. However, inaccurate or entirely erroneous recall did contribute to lower scoring for 132, about 10%, of all items. The remaining items were not recalled at all.

We found that the hypothesized recall-promoting behaviors were uncommon. Agenda setting occurred in only 7 of the 189 visits, while wrap-up occurred in only 27. There were a total of 10 “teach back” events, and only 5 of these happened in resolution processes. Provider open questions occurred in 16 Medical processes and only 4 Behavioral processes. Providers made far more utterances than patients. The ratio of provider to patient utterances was 2.5 in Medical resolution processes and 2.3 in Behavioral resolutions. Finally, OPTION coding found most elements of shared decision making largely absent. For example, “listing options” was observed only 15 times. Pros and cons of multiple options were never discussed in behavioral processes.

### Correlates of recall quality

Recall was strongly associated with patients’ level of formal education (Table 2, p< 0.0001). Only about 38% of resolutions were recalled freely and accurately by people with less than a high school diploma, whereas 65% were recalled freely and accurately by people with a college degree. This difference was highly significant when accounting for clustering within patients, and remained significant when selecting only Medical or only Behavioral resolutions.

**Table 2:** Recall quality of medical and behavioral resolutions and patient level of formal education (N, row % within category).

	Recall Quality: Medical			Recall Quality: Behavioral			Recall Quality: All			
Education	Erroneous or No Recall	Recalled with prompt	Recalled freely and accurately	Erroneous or No Recall	Recalled with prompt	Recalled freely and accurately	Erroneous or No Recall	Recalled with prompt	Recalled freely and accurately	Total
<12^th^ grade	60 (33%)	52 (29%)	69 (38%)	21 (31%)	21 (31%)	25 (37%)	81 (33%)	73 (20%)	94 (38%)	248
12^th^ grade	46 (16%)	112 (38%)	133 (46%)	16 (13%)	60 (47%)	51 (40%)	62 (15%)	172 (41%)	184 (44%)	418
Some college	37 (15%)	94 (38%)	118 (47%)	5 (4%)	47 (39%)	67 (56%)	42 (11%)	141 (38%)	185 (50%)	368
4 yrs. college	12 (6%)	52 (27%)	132 (67%)	2 (2%)	33 (37%)	54 (61%)	14 (5%)	85 (30%)	186 (65%)	285
Total	155 (17%)	310 (43%)	452 (49%)	44 (11%)	161 (40%)	197 (49%)	199 (15%)	471 (36%)	649 (49%)	1,319

P < .0001 for all contrasts by chi square within each category

Patient age, gender, race/ethnicity, and the specialty (nephrology vs. cardiology) were not associated with recall quality in any models (all p>0.05). In the model of behavioral resolutions (Table 3), the interaction between resolution count and patient education was statistically significant (P = 0.03). For patients with less than high school education, more resolutions were associated with poorer recall; the same relationship was not seen for more educated patients. The ratio of provider to total utterances in the resolution process was also significantly associated with recall; a higher ratio was associated with worse recall, (β = -0.04, p = 0.01). Even after accounting for the interaction, there was a main effect of resolution count per visit (β = -0.08, p = 0.04). There was no residual main effect of education (p = 0.34). The only independent variable that was statistically significant in the models of medical resolutions was the ratio of provider to total utterances (β = -0.02, p = 0.02).

**Table 3:** Multivariable ordered logit model predicting patient recall.

	Medical			Behavioral		
	Beta**	SE	p-value	Beta**	SE	p-value
Resolution count x patient education	0.05	0.04	0.20	0.10	0.04	0.03
Provider/total utterances*	-0.02	0.01	0.02	-0.04	0.01	0.01
Patient education†	0.38	0.61	0.53	-0.80	0.83	0.34
Resolution count/visit	-0.03	0.03	0.42	-0.08	0.04	0.04

* This fraction refers to the ratio of provider utterances to total utterances in the resolution process

** Beta can be interpreted as the change in odds ratio of being in a higher category of the dependent variable for each increment of the independent variable

† (1 = ≥12 years, 0 = <12 years)

The interpretation of the coefficients is that if a patient were to have one more resolution in a visit, the ordered log odds of being in the higher resolution quality level would decrease by .055; while the odds would decrease by .028 for each 1% increase in the percentage of utterances by providers. We should note that the proportional odds assumption is not met–the contrast between erroneous or no recall and accurate recall with prompting dominates over prompted vs. unprompted recall. While this complicates interpretation of the coefficients, it does not invalidate the finding of associations.

## Discussion

This study has demonstrated the utility of a structured approach to assessing recall quality, combined with the CASES method to support assessment and analysis of multiple decision making processes within an encounter. To our knowledge, these tools are novel in the information they can generate about interaction processes in clinical decision making. Research has focused on cognitive biases, such as the relationship of gain and loss framing to decision outcomes,[44, 45] and ways of presenting numerical risk constructs.[46] The OPTIONS system assesses the content of the content of clinical decision making communication. However, content is only one dimension of communication. It is the interaction process, as assessed here by CASES and the GMIAS, that determines whether patients and family members understand and remember information, and can participate fully in shared decision making.

We found that there is substantial variation in the number of threads and resolutions per visit. This is presumably a factor in the cognitive and decisional burden on patients. We found using rigorously structured inquiry that patients in this sample recalled about 85% of items accurately, but that less educated patients recalled fewer, particularly when confronted with a large number of items. We also found that patients recall fewer items when physician utterances are more numerous compared to patient utterances (a measure called “verbal dominance” in the literature), which suggests a relationship between patient engagement and patient recall.

Our finding that 85% of items are recalled accurately, with or without prompting, represents a higher rate of recall than in most studies. Not all reports of previous studies make it clear whether prompting was used, nor do they clarify how accuracy was operationalized, so we cannot say how comparable this is to previous observations. We also cannot say to what extent items recalled only with prompting would have been remembered later by respondents if we had not done the interview; nor can we say how much information would have been retained at longer-term follow-up. These are appropriate questions for further research.

There is no standard for what percentage of decisions and recommendations patients ought to remember accurately. Despite our finding of a relatively high percentage of accurate recall, the 15% of items that are not remembered may be consequential. In our sample, they include, for example, instructions to reduce salt intake, to change medication dosages, and to seek medical attention if certain symptoms occur. Better understanding of the prevalence of risk and harm to patients from inaccurate recall is needed.

We find that patient recall of specific treatment decisions and behavioral recommendations is relatively poor for people with little formal education; that recall is negatively associated with a higher ratio of provider to patient talk in resolution processes; and that the association of patient education with recall is moderated by the number of items to be recalled.

We also found that recall-promoting behaviors by providers are rare, as are elements of shared decision making. We must note, however, that with an average of nearly 8 decision items per visit, it would probably not be feasible to have a substantial shared decision process for all of them; nor would patients necessarily expect or want this. Most decisions in these visits are routine and, at least from the physician’s point of view do not present difficult tradeoffs. Typical items include test ordering, dietary restrictions, or medication adjustment. Of course these might be far more problematic for the patient than the physician realizes. A challenge for future research is to determine what apparently minor choices or instructions might actually present significant trade-offs for patients, and how providers and patients can determine what choices call for substantial decisional processing.

We are not the first investigators to observe that there has been surprisingly little change in physician-patient interaction over the decades.[47] While our study does not provide any particular insight into why this is true, it serves as confirmation. It also points to the greater challenge of achieving a consistently high level of patient understanding and recall with less educated patients.

The consensus of the symposium participants (see 2.6, “stakeholder engagement”) was that physicians generally want to practice more patient-centered medicine, but that time pressure is a major obstacle. Agenda setting, teach back, discussion of multiple options, and open questions encouraging more patient talk are all perceived as taking up scarce time. The conclusion was that simply training physicians to use these techniques is unlikely to produce major change. What is needed is reform and reorganization of the medical enterprise to create more room within the precious time of the physician-patient encounter to adequately process a limited number of therapeutic decisions. Systems for pre-visit planning, putting more medical education and counseling responsibilities on non-physician providers, using technology to facilitate exchange of information and recall, were all among the proposals.

This study is limited to 3 practices, all located in an academic medical center, and a fairly small number of physicians. These are not primary care practices, so it will be important to extend similar work to primary care and other settings. The findings, while based on hypotheses, represent the first time these specific methods have been used and must be considered subject to replication based on larger and more diverse samples of practices.

The study succeeded in showing the feasibility and face validity of the methods, as the findings are intuitively plausible and generally consistent with previous observations. We hope they suggest some ways forward to improving medical interactions and patient-centered outcomes.

Findings suggest that patient recall could be enhanced if providers were to use more of the techniques to encourage patient engagement, such as open questioning, agenda setting, and teach-back; and limit the amount of information to be remembered in a single visit based on an assessment of patients’ ability to recall.

## Supporting information

S1 Text (DOCX)

S2 Text (DOCX)

S3 Text (DOCX)

S4 Text (DOCX)

S5 Text (DOCX)

S6 Text (DOCX)

S7 Text (DOCX)

S8 Text (DOCX)

S9 Text (DOCX)
