# Supplementary material for: Factors associated with patient recall of key information in ambulatory specialty care visits: Results of an innovative methodology
Source: PLoS One. 2018 Feb 1;13(2):e0191940. doi: 10.1371/journal.pone.0191940 (PMC5794108; doi:10.1371/journal.pone.0191940)
Supplement: S1 Text — (DOCX) [file pone.0191940.s001.docx]

**Methods for Analysis of Decision-Related Communication in Outpatient Care**

**Provider Demographic Questionnaire**

1. Name: ________________________

2. Gender (circle one): Male Female

3. Year of birth: ______

4. What type of health care provider are you? (circle one):

1. Attending physician
2. Fellow
3. Resident
4. Intern
5. Medical student
6. Nurse practitioner
7. Physician assistant
8. Registered nurse
9. Other: ________________________

**For physicians,**

5. When did you receive your degree? : ________________________

6. When did you complete your residency (if applicable)? : ________________________

7. When did you complete your fellowship (if applicable)? : ________________________

8. When did you get board certified (if applicable)? : ________________________

**For non-physicians,**

9. When did you receive your certification? : ________________________
