# Supplementary material for: Factors associated with patient recall of key information in ambulatory specialty care visits: Results of an innovative methodology
Source: PLoS One. 2018 Feb 1;13(2):e0191940. doi: 10.1371/journal.pone.0191940 (PMC5794108; doi:10.1371/journal.pone.0191940)
Supplement: S2 Text — (DOCX) [file pone.0191940.s002.docx]

**Physician-Patient Communication, Decision Making and Management in Chronic Pain**

**Patient Demographic Questionnaire**

**Q1:** What is your sex or gender?

Male [ ] 1

Female [ ] 2

Transgender [ ] 3

Don’t know [ ] 8

Refused [ ] 9

**Q2:** What is your date of birth? ___ ___ ___

Don’t know [ ] 98

Refused [ ] 99

**Q3:** How would you describe your race or ethnic group?

White, not Hispanic [ ] 1

African-American [ ] 2

Haitian [ ] 3

English speaking Caribbean [ ] 4

Cape Verdean [ ] 5

Latina/o/Hispanic [ ] 6

Asian [ ] 7

Other [ ] 8

Specify: ______________________

Don't know [ ] 98

Refused [ ] 99

**Q4:** In what country were you born?

Continental U.S. [ ] 1 => **GO TO Q6**

Mexico [ ] 2

Puerto Rico [ ] 3 => **GO TO Q5**

Canada [ ] 4

Dominican Republic [ ] 5

Cuba [ ] 6

El Salvador [ ] 7

Guatemala [ ] 8

Colombia [ ] 9

Ecuador [ ] 10

Brazil [ ] 11

Haiti [ ] 12

English Caribbean [ ] 13

Honduras [ ] 14

Nicaragua [ ] 15

Italy [ ] 16

Russia [ ] 17

Spain [ ] 18

China [ ] 19

Ireland [ ] 20

England [ ] 21

Costa Rica [ ] 22

Cape Verde [ ] 23

Other [ ] 24

Specify:________________________

Don’t know [ ] 98

Refused [ ] 99

**Q5:** In what year did you first come to the continental United States (50 states) to live?

Year ___ ___ ___ ___

Don’t know [ ] 98

Refused [ ] 99

**Q6:** What is the highest grade or year of school you have completed or are currently enrolled in? **(CODE ONLY ONE.)**

Never attended school [ ] 0

1st Grade [ ] 1

2nd Grade [ ] 2

3rd Grade [ ] 3

4th Grade [ ] 4

5th Grade [ ] 5

6th Grade [ ] 6

7th Grade [ ] 7

8th Grade [ ] 8

9th Grade [ ] 9

10th Grade [ ] 10

11th Grade [ ] 11

12th Grade [ ] 12

1 Year College [ ] 13

2 Years College [ ] 14

3 Years College [ ] 15

4 Years College [ ] 1

1 Year Graduate [ ] 17

2 Year Graduate [ ] 18

3 Year Graduate [ ] 19

4 Year Graduate [ ] 20

Don't know [ ] 98

Refused [ ] 99
