# Supplementary material for: Factors associated with patient recall of key information in ambulatory specialty care visits: Results of an innovative methodology
Source: PLoS One. 2018 Feb 1;13(2):e0191940. doi: 10.1371/journal.pone.0191940 (PMC5794108; doi:10.1371/journal.pone.0191940)
Supplement: S4 Text — (DOCX) [file pone.0191940.s004.docx]

MEDICC Second/Third Follow-up Interview

Participant ID#_____

Encounter #___

Interviewer____________

Interview Date__________ Start time________

Comments:

[The following need not be read verbatim but essential elements should be included.]

Good [afternoon] Mr/Mrs/Ms [name], this is [interviewer name] with the MEDICC study. You’ll remember I was going to call you today to ask about your visit a few days ago with Dr. [name]. Is this a good time to talk?

[If not, reschedule; see case control form]

So how are you today?

Okay, as you’ll remember I want to record our phone call to make sure we have an accurate record of what you say. Again, it’s completely confidential and only a few people connected with the study will hear the recording. We’ll edit out any information from the transcript that might identify you. Do you have any questions? Are you ready to begin?

Okay, the recorder is on now.

1: Background and general experience of the visit

**Q1_1:** So tell me about your visit with Dr. [name] last [day of week].

*Probe if necessary:*

**Q1_1a:** What did you like about it?

**Q1_1b:** What did you not like about it?

**Q1_1c:** Did you have to wait a long time?

**Q1_1d:** Did you like the way you were treated by the clinic staff?

**Q1_2**: What is your health like now?

**Q1_2a:** Have you had any symptoms as a result of [heart disease/kidney disease]?

**Q1_2ai**: What are those symptoms? How do they affect your life?

**Q1_3**: How do you feel emotionally?

**Q1_3a**: Are you taking any medications for emotional problems?

**Q1_3b:** Are you getting any counseling?

**2: The previous visit**

Okay, you saw Dr. [name] last [day of the week], right?

**Q2_1:** In addition to Dr. [name], did you see any other health care providers in the clinic that day, such as another doctor, a nurse, or any other kind of professional?

**Q2_1a: [if yes]** Who were these people? Can you tell me the person’s name and what kind of practitioner they are?

**Q2_1b: [for each individual named]:** About how long did you spend with [this person]? How satisfied were you with your interaction with [this person]?

**Q2_2:** Okay, now I’m going to ask you about some of the specific things that happened in your visit with Dr. [name] and [other names if any]. What information did Dr. [name] or other people give you about [heart disease/kidney disease] or your particular condition?

[Note: It is possible that treatment decisions, recommendations or prescriptions may be volunteered here. Say, “Oh that’s great, I want to talk about treatment and prescriptions a bit later. First let’s just talk about any information you got*.*”] [Use open probes such as “what else do you remember?” or “anything else” until respondent has nothing further to volunteer.]

**Q2_2a:** [For each item mentioned, if respondent saw more than one provider, ask] And do you remember who told you this? Was it Dr. [name] or somebody else?

**Q2_2b: [**For each item mentioned] [Ask open questions to elicit respondent’s full explanation of the concept, such as “can you tell me a little more about that”? Or “Can you tell me why that is important?]

**Q2_3:** [Go to the structured abstract.* For each informational item respondent did not volunteer, ask specifically] Do you remember talking about [item]?

**Q2_3a:** [If yes] And do you remember who you talked with about this? Was it Dr. [name] or somebody else?”

**Q2_3b:** [For each item mentioned] [Ask open questions to elicit respondent’s full explanation of the concept, such as “can you tell me a little more about that”? Or “Can you tell me why that is important?]

**Q2_4:** On the whole, did you feel that you got the information you wanted during this visit? Were there any questions you had that you didn’t get answers to? Is there anything you wish you understood better?

*See attachment.

**Q2_4:** Now I’d like to talk about any decisions, recommendations, prescriptions or options that were discussed during the visit about medical treatment or the best way to take care of yourself. What treatments or recommendations did you talk about during the visit?

**Q2_4a:** [For each item mentioned, if respondent saw more than one provider, ask] And do you remember who discussed this with you? Was it Dr. [name] or somebody else?

**Q2_4b: [**For each item mentioned] [Ask open questions to elicit respondent’s full explanation of the concept, such as “can you tell me a little more about that”? Or “Can you tell me why that is important?]

**Q2_4c:** [If appropriate] And what did you decide? Are you going to [take the medication, get more exercise, quit smoking . . . ]

**Q2_4d:** [For any prescriptions respondent received]. Have you filled the prescription? Are you taking the medication?

**Q2_4di:** What did Dr. [name] tell you about this medication? Why should you take it? What will it do for you? What side effects should you watch out for? What schedule are you supposed to take it on?

**Q2_4e:** [For each item mentioned] What do you like or not like about this [treatment/lifestyle change/option]?

**Q2_5:** **:** [Go to the structured abstract.* For each informational item respondent did not volunteer, ask specifically] Do you remember talking about [item]?

**Q2_5a:** [If yes] And do you remember who you talked with about this? Was it Dr. [name] or somebody else?”

**Q2_5b:** [For each item mentioned] [Ask open questions to elicit respondent’s full explanation of the concept, such as “can you tell me a little more about that”? Or “Can you tell me why that is important?]

**Q2_5c:** [If appropriate] And what did you decide? Are you going to [take the medication, get more exercise, quit smoking . . . ]

**Q2_5d:** [For any prescriptions respondent received]. Have you filled the prescription? Are you taking the medication?

**Q2_5di:** What did Dr. [name] tell you about this medication? Why should you take it? What will it do for you? What side effects should you watch out for? What schedule are you supposed to take it on?

**Q2_5e:** [For each item mentioned] What do you like or not like about this [treatment/lifestyle change/option]?

**Q2_6:** So how would you describe the process of making these decisions? Who makes decisions about treatment? You, your doctor, you and your doctor together, or what?

**Q2_6a**: Is this what you want? Are you satisfied with the process?
