# Supplementary material for: Factors associated with patient recall of key information in ambulatory specialty care visits: Results of an innovative methodology
Source: PLoS One. 2018 Feb 1;13(2):e0191940. doi: 10.1371/journal.pone.0191940 (PMC5794108; doi:10.1371/journal.pone.0191940)
Supplement: S5 Text — (DOCX) [file pone.0191940.s005.docx]

|  | Disagree Strongly | Disagree | Agree | Agree Strongly | *Refused* | *Don’t know* |
| --- | --- | --- | --- | --- | --- | --- |
| PAM1  a. When all is said and done, I am the person who is responsible for managing my health condition. | 1 | 2 | 3 | 4 | *98* | *99* |
| PAM2  b. Taking an active role in my own health care is the most important factor in determining my health and ability to function. | 1 | 2 | 3 | 4 | *98* | *99* |
| PAM3  c. I am confident that I can take actions that will help prevent or minimize some symptoms or problems associated with my health condition. | 1 | 2 | 3 | 4 | *98* | *99* |
| PAM4  d. I know what each of my prescribed medications do. | 1 | 2 | 3 | 4 | *98* | *99* |
| PAM5  e. I am confident that I can tell when I need to go get medical care and when I can handle a health problem myself. | 1 | 2 | 3 | 4 | *98* | *99* |
| PAM6  f. I am confident I can tell my health care provider concerns I have even when he or she does not ask. | 1 | 2 | 3 | 4 | *98* | *99* |
| PAM7  g. I am confident that I can follow through on medical treatments I need to do at home. | 1 | 2 | 3 | 4 | *98* | *99* |
| PAM8  h. I understand the nature and causes of my health condition(s). | 1 | 2 | 3 | 4 | *98* | *99* |
| PAM9  i. I know the different medical treatment options available for my health condition. | 1 | 2 | 3 | 4 | *98* | *99* |
| PAM10  j. I have been able to maintain the lifestyle changes for my health that I have made. | 1 | 2 | 3 | 4 | *98* | *99* |
| PAM11  k. I know how to prevent further problems with my health condition. | 1 | 2 | 3 | 4 | *98* | *99* |
| PAM12  l. I am confident I can figure out solutions when new situations or problems arise with my health condition. | 1 | 2 | 3 | 4 | *98* | *99* |
| PAM13  m. I am confident that I can maintain lifestyle changes like diet and exercise even during times of stress. | 1 | 2 | 3 | 4 | *98* | *99* |
