# Supplementary material for: Factors associated with patient recall of key information in ambulatory specialty care visits: Results of an innovative methodology
Source: PLoS One. 2018 Feb 1;13(2):e0191940. doi: 10.1371/journal.pone.0191940 (PMC5794108; doi:10.1371/journal.pone.0191940)
Supplement: S6 Text — (DOCX) [file pone.0191940.s006.docx]

**“Experience with Your Doctor Survey” [Modified]**

**Managing Your Care**

1. [15] In the last visit, how often did your doctor explain things in a way that was easy to understand?

- Never
- Almost never
- Sometimes
- Usually
- Almost always
- Always

1. [16] In the last visit, how often did your doctor listen carefully to you?

- Never
- Almost never
- Sometimes
- Usually
- Almost always
- Always

1. [17] In the last visit, did you talk with your doctor about any health problems or symptoms that were bothering you?

- Yes
- No 🡪 If No, Go to Question 20

1. [18] In the last visit, how often did your doctor give you clear instructions about what to do to take care of the health problems or symptoms that were bothering you?

- Never
- Almost never
- Sometimes
- Usually
- Almost always
- Always

1. [19] In the last visit, how often did your doctor give you clear instructions about what to do if your symptoms got worse or came back?

- Never
- Almost never
- Sometimes
- Usually
- Almost always
- Always

1. [21] In the last visit, how often did your doctor seem to know all the important information about medical history?

- Never
- Almost never
- Sometimes
- Usually
- Almost always
- Always

1. [22] In the last visit, how often did your doctor show respect for what you had to say?

- Never
- Almost never
- Sometimes
- Usually
- Almost always
- Always

1. [23] In the last visit, how often did your doctor spend enough time with you?

- Never
- Almost never
- Sometimes
- Usually
- Almost always
- Always

1. [24] How would you rate your doctor’s knowledge of you as a person, including values and beliefs that are important to you?

- Very poor
- Poor
- Fair
- Good
- Very good
- Excellent

1. [27] In the last visit, did you and your doctor talk about things in your life that worry you or cause you stress?

- Yes, definitely
- Yes, somewhat
- No, definitely not

1. [28] In the last visit, did your doctor ask whether there was a period of 2 weeks or more where you felt sad, empty or depressed?

- Yes
- No

**Overall Rating**

1. [35] Using any number from 0 to 10, where 0 is the worst doctor possible and 10 is the best doctor possible, what number would you use to rate your doctor?

- 0 Worst doctor possible
- 1
- 2
- 3
- 4
- 5
- 6
- 7
- 8
- 9
- 10 Best doctor possible

1. [36] Would you recommend your doctor to your family and friends?

- Definitely yes
- Probably yes
- Not sure
- Probably not
- Definitely not

**About You**

1. [44] In general, how would you rate your overall health?

- Excellent
- Very good
- Good
- Fair
- Poor
