# Supplementary material for: Factors associated with patient recall of key information in ambulatory specialty care visits: Results of an innovative methodology
Source: PLoS One. 2018 Feb 1;13(2):e0191940. doi: 10.1371/journal.pone.0191940 (PMC5794108; doi:10.1371/journal.pone.0191940)
Supplement: S7 Text — (DOCX) [file pone.0191940.s007.docx]

**MEDICC CASES coding scheme**

**Inductions**

**1.0** Request

**2.0** Problem presentation

2.1 New symptoms / condition / action

2.2 Already known symptoms/condition / action

**3.0** Inquiries

3.1 Focused question

3.2 Invitation question

**4.0** Cued

**4.1** Cued by interlocutor

**4.11** Medicalizing something interlocutor said

**4.2** Test results

**4.21** Bad results requiring resolution

**4.22** Good results not requiring resolution

**4.3** Standard checklist

**4.4** Exam finding

**4.5** Pre-agenda topic

**4.51** “The nurse told me you wanted to talk about”

**5.0** Narrative without identified problem

**83** Coder doesn’t know

**Processes**

**P** Patient history

**S** Investigation

**M** Resolution – Medical intervention

**B** Resolution – Behavioral counseling

**I** Information

**E** Engage feelings

**A** Agenda setting

**Processes that aren’t threads:**

**31** Socializing / ritual / levity

**32** Trash

**33** Logistics (age, phone number, address, etc.)

**34** Wrap up

**76** Below thread D-D interactions

**77** Check list questions

**78** Physical exam

**Resolutions**

**1.0** **Information**

**1.1** Diagnosis

**1.11** Non-problematic diagnosis (requires no further action)

**1.12** Non-problematic test results

**1.2** Instruction on ***how to do*** something

**1.3** All other information, explanation or instruction

**2.0** **Treatment recommendation (prescribing / procedure / behavior change)**

**2.1** Prescribing

**2.11** New prescription

**2.111** Patient commissive

**2.112** Patient refusal

**2.12** Medication change/maintenance

**2.121** Patient commissive

**2.122** Patient refusal

**2.2** Procedure

**2.21** Patient commissive

**2.22** Patient refusal

**2.3** Behavior change/maintenance

**2.31** Patient commissive

**2.32** Patient refusal

**2.4** Self-care

**2.41** Patient commissive

**2.42** Patient refusal

**2.5**– Ordering tests/ test maintenance

**2.51** Patient commissive

**2.52** Patient refusal

**2.6** Decision Deferred (e.g., until test results)

**2.7** Wait-and-see” approach

**3.0 Referral**

**3.1** Referral to other doctor

**3.2** Referral to website or other reading material

**3.3** Referral to some kind of program

**4.0** **Provider’s action**

**4.1** Doctor commissive or promise

**4.2** Doctor does something there and then

**4.3** Offer

**4.4** Probably resolved but we don’t have clear verbal

**4.5** Resolved with empathy

**5.0** **No need to be resolved (not a problem)**

**5.1** Patient doesn’t see it as a problem

**6.0** **Not resolved**

**6.1** because nothing can be done

**6.2** because doctor dropped the ball

**9.0** **Unattached resolution** (doctor gives advice without a problem being clearly stated)

**9.1** We don’t know the problem

**9.2** We know patient does not have problem being solved

**9.3** Patient denies the fact

**83** Coder doesn’t know 11/18/2014
