# Supplementary material for: Factors associated with patient recall of key information in ambulatory specialty care visits: Results of an innovative methodology
Source: PLoS One. 2018 Feb 1;13(2):e0191940. doi: 10.1371/journal.pone.0191940 (PMC5794108; doi:10.1371/journal.pone.0191940)
Supplement: S8 Text — (DOCX) [file pone.0191940.s008.docx]

### CODING MANUAL FOR

**MEDICC FOLLOW-UP MAPPING**

Department of Health Services, Policy and Practice, Brown School of Public Health

##### July 2015

**INTRODUCTION: THE FOLLOW-UP INTERVIEW**

The follow-up interview is both a transcribed and audio semi-structured interview about a patient’s experience in a recent, corresponding medical encounter. It is conducted by a research assistant on a consented, participating patient of one of the three clinics involved in the MEDICC project. There’s a wealth of qualitative and quantitative data on the corresponding medical encounter in these interviews, and as part of the data-collection and organization portion of the MEDICC project this data needs to be “mapped” back onto their corresponding medical encounters. We have four sets of data that each have a different data storage system:

- **Patient Demographic Data**, which is stored in one Excel file. This is completed and ready for analysis.
- **Structured Interview Responses**, which are stored in one Excel file. This is completed and ready for analysis.
- **Process-Specific Data** is that data which relates to the specific topics being discussed in a medical encounter. These are to be mapped back onto completely coded IA transcripts in Excel form for analysis.
- **Case-Specific Data** is that data which relates to the patient and their history as a whole. This is to be coded into a Filemaker database by patient-provider dyad, which will then be exported as an Excel file for further analysis.

This manual is meant to explain how to code **Process-Specific Data** and **Case-Specific Data**.

**PROCESS-SPECIFIC DATA MAPPING**

1. To begin, open a completely coded (parsed, SA coded, topic coded, CASES coded, and OPTION coded) transcript in IA.
2. Export it to the appropriate folder in P:\gmias\shared\MEDICC\Data\Coded csv files.
3. There, copy and paste it to the appropriate folder for follow-ups in P:\gmias\shared\MEDICC\Data\Coded_f-ups.
4. Open the file in Excel. Add six additional columns in the Excel spreadsheet between the “Resolution” and “Option” columns. These six columns should be named:

- **Recall**
- **Speaker recall**
- **Understanding** for information and investigation processes only
- **Follow-through** for resolution processes only
- **Reasoning Recall** for resolution processes only
- **Reasoning Understanding** for resolution processes only
- Use the “Hide” function on extraneous columns of the interview in Excel as you see fit in order to make coding easier. The topic codes, CASES thread, process and resolution columns are the most pertinent to follow-up coding.

1. Using the transcript of the follow-up interview, go through the encounter and code using the system below. Be sure to place codes at what you perceive as the *last* utterance of distinct parcels of Investigation, Information, or Resolution processes. For resolution processes, this means the follow-up codes will go in the same utterances as the resolution codes if the CASES coding was completed correctly. For Investigation and Information processes, this means the follow-up codes will go in the last utterances of a topic-code’s connection to S or I processes. If there any distinct parcels of Investigation, Information, or Resolution processes that are not brought up by either the interviewer or interviewee in the follow-up interview, they are to be coded “81” in the Recall column.
   1. Different providers receive different sets of Investigation and Information follow-up codes, even if they are discussing the same results. If a patient recalls discussing Investigation or Information with one provider and not the other, both providers’ discussions receive Recall and Understanding codes, but only one would receive an affirmative Speaker Recall code.
   2. In the rare cases that Investigation or Information processes are discussing different sets of findings while using the same topic code (for findings that do not have their own specific topic codes, such as stress tests and catheterizations for heart disease [both 1.13], or for kidney disease versus hypertension information for kidney disease[both 1.1]), the coder should use their own discretion as to the last utterance of discussion of each specific set of findings or information.
   3. Exceptions to follow-up coding next to resolution codes are the 5.x and 6.x resolution codes, since they denote no completed resolutions taking place in the thread.
2. Once coding is complete, “Save As” the edited file as a .csv file, with “_fu_” added to the file name, so as to resemble this: “HFC0XP00X_v_1_fu_NM”.
3. Continue on to Case-Specific data mapping.

**RECALL (0-1-2)**

In recall for Resolutions, code only when there is a resolution code present.

In recall for all other information-- S and I processes-- code at the last utterance of what one perceives to be a discrete amount of investigation/information.

In Recall, **0** stands for "does not recall" and "does not recall with prompt". This means that any Investigation or Information process that is not justifiably covered by the patient's response in the semi-structured follow-up interview can be marked as 0.

**1** stands for "recalled with prompt". The interviewer in the semi-structured follow-up has to mention the item or information before the patient in order for this to apply.

**2** stands for “freely recalled”, meaning the patient, without mention of the item or information by the interviewer, brings up the topic in the semi-structured interview.

The **81** code represents that the follow-up interviewer did not discuss the information or resolution despite it being discussed in the doctor-patient encounter.

**SPEAKER RECALL (0-1)**

In addition to normal recall in encounters where there’s more than one provider, Speaker Recall must be coded. **0** stands for not being able to remember which provider the information that a person recalls came from; **1** stands for accurately recalling which provider communicated the information or resolution.

**UNDERSTANDING (0-1-2)**

Understanding codes are only applied to Investigation and Information processes.

In Understanding codes, **0** stands for "little to no understanding" and "incorrect understanding". For example, a patient recalls freely that the doctor mentioned the results of her most recent echocardiogram. However, she incorrectly believes she has a small blockage, which was directly refuted by the doctor in the encounter. In this case, the utterances involved in explaining that the patient's ECHO did not show an arterial blockage would receive a recall code of 2 and an understanding code of 0.

**1** stands for "basic understanding" or "ballpark understanding", where the basic concepts are grasped by the patient but particulars are misunderstood. In HFC01P049_v_1, the patient recalls with a prompt that the doctor mentioned she was a low-risk patient in terms of her heart, but when asked about what percentage risk she was placed in by her doctor she responded 5%. In the encounter, the doctor mentions that she has 1% risk of further cardiac complications in the near future. Thus the processes involved with the patient's risk of serious cardiac complications would receive a recall code of 1 and an understanding code of 1.

**2** stands for "correct understanding" or "thorough understanding", for when the patient accurately articulates what the doctor mentioned during the encounter.

Any Investigation or Information processes that are not recalled or denied after prompting, thus coded 0, are not given understanding codes. It's assumed that a patient cannot understand something they cannot remember or deny having occurred.

**FOLLOW-THROUGH (0-1)**

Though this is not the case with all follow-up interviews and only applies to resolution processes, if the interviewer asks whether or not the patient carries out a suggested resolution, this code can be used to mark it in the recall column. **0** represents that the patient *did not* follow through on the resolution (i.e. didn’t fill out their prescription, missed their follow-up appointment, declined dialysis, etc), while **1** represents that the patient acknowledges that they did follow through on the resolution.

**REASONING RECALL (0-1)**

This measure is of whether the patient, in their recollection of a resolution process in the interview, *recalls* the reasoning of why a certain resolution was decided upon for a given issue in the medical encounter. A **0** represents not being able to recall why, and a **1** represents being able to recall why, *regardless of whether that reasoning is correct or incorrect*.

**REASONING UNDERSTANDING (0-1-2)**

If the reasoning of a resolution process is able to be recalled, then the coder must also code the patient’s understanding of the reasoning behind a decision made in the medical encounter. This aspect mirrors the basic understanding score: **0** stands for erroneous understanding, **1** stands for partial or partially incorrect reasoning, and **2** stands for correct understanding of the reasoning behind a decision.

**CASE-SPECIFIC DATA MAPPING**

Open the MEDICC_FUMAP_NM_1 Filemaker database. If you’re coding the first visit of a doctor-patient dyad, start a new record. If you’re coding the second or higher visit of a doctor-patient dyad, find the initial visit, then navigate to the appropriate-numbered-visit’s layout using the drop-down layout menu on the bottom left of the Filemaker navigation bar.

Using the follow-up interview, fill in the answers to as many of the questions posed in the database’s record as you can. If the answer is not to be found in the follow-up interview, leave the question unanswered. Use the “Notes” section to put in any abnormalities with the case-specific portions of the follow-up interview.
