# Supplementary material for: Factors associated with patient recall of key information in ambulatory specialty care visits: Results of an innovative methodology
Source: PLoS One. 2018 Feb 1;13(2):e0191940. doi: 10.1371/journal.pone.0191940 (PMC5794108; doi:10.1371/journal.pone.0191940)
Supplement: S9 Text — (DOCX) [file pone.0191940.s009.docx]

*USE THE CODES AT THE START*

1. **Draws attention to a problem requiring decision making process (Dr/Pt)**

**1.0** Not observed

**1.1** Observed P, S, M (MOSTLY F/U), B

1. **States the existence of more than one option to deal with the problem (Dr/Pt)**

**2.0** Not observed

**2.1** Observed M, B

1. **Pt’s preferred approach to receiving information (Dr/Pt)**

**3.0** Not observed

**3.1** Dr. offers a closed question

**3.2** Dr. offers Pt. an open choice of how to approach the flow of information

**3.3** Pt. presents their preferred approach to receiving information

I, M, B

1. **Lists options (including “no action”) (Dr/Pt)**

**4.0** Not observed

**4.1** Observed

M, B

1. **Pros and cons of options (Dr/Pt)**

**5.0** No explanation

**5.1** Pros and cons of a single option

**5.2** Pros and cons of most of available options

M, B

1. **Pt’s expectations about how to manage the problem (Dr/Pt)**

**6.0** Not observed

**6.1** No f/u or elaboration by the interlocutor

**6.2** Open questions on either side, broad conversation

B, M, I

1. **Pt’s fears or concerns about how to manage the problem (Dr/Pt)**

**7.0** Not observed

**7.1** Acknowledgement w/o elaboration

**7.2** Acknowledgement with elaboration

B, M, E

1. **Checks Pt’s understanding (Dr)**

**8.0** Not observed

**8.1** Simple CQ (Okay? Alright?)

**8.2** Closed, non-specific knowledge-checking question

**8.3** Teach back question

EVERYWHERE

1. **Offers opportunities to ask questions (Dr)**

**9.0** Not observed

**9.1** Closed question

**9.2** Open question

EVERYWHERE

1. **Pt’s preferred role (active or passive) in decision making (Dr/Pt)**

**10.0** Not observed

**10.1** Observed

M, B

1. **Indicates the need for a decision making or deferring state (Dr/Pt)**

**11.0** Not observed

**11.1** Observed

M, B

1. **Indicates the need to review the decision or deferment (Dr/Pt)**

**12.0** Not observed

**12.1** Need is implied

**12.2** Need is explicitly stated

**M, B**
